# Supplementary material for: Collective dynamics and long-range order in thermal neuristor networks
Source: Nat Commun. 2024 Aug 14;15:6986. doi: 10.1038/s41467-024-51254-4 (PMC11324871; doi:10.1038/s41467-024-51254-4)
Supplement: Supplementary file 1 — Supplementary Information [file 41467_2024_51254_MOESM1_ESM.pdf]

Supplementary Information: Collective dynamics and long-range order in thermal neuristor networks

Yuan-Hang Zhang,[1,*] Chesson Sipling,[1] Erbin Qiu,[1,2] Ivan K. Schuller,[1] and Massimiliano Di Ventra[1,†]

[1] *Department of Physics, University of California San Diego, La Jolla, CA 92093*
[2] *Department of Electrical and Computer Engineering, University of California San Diego, La Jolla, CA 92093*

Appendix A: Emergence of long-range order

In this section, we delve deeper into Eqs. (1) and (2) in the main text to explore the emergence of long-range order (LRO). In particular, we show that it arises due to the time non-local (memory) response of the system.

Heuristically, the relatively slowly varying temperature field gradually couples spatially separated neuristors, giving rise to long-range correlations. This intuition suggests that only terms in these equations that couple V_i and T_i or introduce thermal diffusive coupling are necessary to induce LRO. As our analysis continues, it will become more clear that these are, in fact, the only terms that are necessary to make the existence of LRO manifest. For clarity, we rewrite Eqs. (1) and (2) from the main text below, with "..." representing the terms in the original equations which are now irrelevant for the purposes of our analysis:

$$\begin{aligned} \dot{V}_i &= \frac{-V_i}{\tau_{V_i}} + \dots , \\ \dot{T}_i &= \frac{1}{\tau_{\mathrm{T}}}\left(\frac{V_i^2}{S_{\mathrm{c}}R_i} + \nabla^2 T_i\right) + \dots . \end{aligned} \tag{A1}$$

Here, $\tau_{V_i} \equiv CR_i$ represents a site-dependent voltage timescale, and $\tau_{\mathrm{T}} \equiv C_{\mathrm{th}}/S_{\mathrm{c}} \approx 50\tau_{\mathrm{th}}$ is the intersite thermal diffusion timescale. The latter differs from τ_{th} by a factor of about 50, as the thermal coupling between sites is significantly weaker than with the environment. To simplify our analysis, we assume $\tau_{V_i}^{-1}$ is a linear interpolation between the inverse timescale in the insulating state (τ_{ins}^{-1}) and the metallic state (τ_{met}^{-1}), across a range within the hysteresis loop (see Fig. 1(b) in the main text), governed by the following equations:

$$\frac{1}{\tau_{V_i}} = \frac{\alpha(T_i)}{\tau_{\mathrm{ins}}} + \frac{1-\alpha(T_i)}{\tau_{\mathrm{met}}} , \tag{A2}$$

$$\alpha(T_i) = \frac{1}{2}\left(1 - \left(\frac{T_i - T_{\mathrm{c}}}{w}\right)\right) . \tag{A3}$$

Here, T_{c} and w characterize the center and width of the hysteresis loop, respectively.

This approximation, which defines $\alpha(T_i)$, is inspired by Matthiessen's rule [1], traditionally applied in scattering contexts. By focusing solely on T_i-dependent terms in $\tau_{V_i}^{-1}$, we explicitly couple the current and thermal dynamics without reference to R_i:

$$\begin{aligned} \dot{V}_i &= \frac{1}{\tau_V}\left(\frac{-T_i V_i}{T_{\mathrm{c}}}\right) + \dots , \\ \dot{T}_i &= \frac{1}{\tau_T}\left(\zeta T_i V_i^2 + \nabla^2 T_i\right) + \dots . \end{aligned} \tag{A4}$$

In (A4), $\tau_V^{-1} \equiv \frac{T_{\mathrm{c}}(\tau_{\mathrm{ins}} - \tau_{\mathrm{met}})}{2w\tau_{\mathrm{ins}}\tau_{\mathrm{met}}} \approx \frac{T_{\mathrm{c}}}{2w\tau_{\mathrm{met}}}$ (since $\tau_{\mathrm{ins}} \gg \tau_{\mathrm{met}}$) and $\zeta \equiv \frac{C}{2w\tau_{\mathrm{met}}S_{\mathrm{c}}}$. Additionally, we confirm that both $T_i/T_{\mathrm{c}} \sim 1$ and $\zeta V_i^2 \sim 1$ with our chosen parameter values (see Table I in the main text, and note that $V_i \sim$ 5V during spiking dynamics). Therefore, τ_V and τ_T are representative *site-independent* timescales of the relevant current and thermal dynamics, respectively. Given our chosen parameter values, $\tau_V \sim 10$ ns and $\tau_T \sim 10$ μs, indicating a separation of timescales.

Considering these characteristic timescales τ_V and τ_T, we can treat the contributions to $\dot{T}_i$ written in (A4) as small over intervals $\Delta t \lesssim \tau_T$. Thus, the relevant contributions to T_i over each such interval will be approximately constant. Although experiment/simulation suggests a more comprehensive thermal timescale might be shorter (the inter-spike interval is $\sim 1\mu$s), recall that we are only considering terms that couple T_i and V_i or introduce diffusive coupling. While T_i does implicitly depend on voltage coupling, thermal coupling with adjacent sites and the environment, *and* noise, contributions to T_i which only depend on the time-evolution terms kept in Eq. (A4) will always exist. For clarity, we call these relevant contributions $\tilde{T}_i$ and $\tilde{V}_i$.

We now show that by iteratively integrating $\tilde{T}_i$ and $\tilde{V}_i$ over a prolonged period of time, the presence of long-range current couplings becomes manifest. This "time coarse-graining" approach is analogous to methods used in other complex systems studies [2, 3].

First, we evolve $\tilde{T}_i$ over intervals of length τ_T, during which $\tilde{T}_i$ is approximately constant:

$$\begin{aligned} \tilde{T}_i(\tau_T) &= T_i(0) + \int_0^{\tau_T} \dot{\tilde{T}}_i(t)dt \\ &\approx \left(1 + \zeta\overline{V_{i,1}^2} + \nabla^2\right)T_i(0) \equiv \hat{L}_0 T_i(0) , \end{aligned} \tag{A5}$$

* email: yuz092@ucsd.edu
† email: diventra@physics.ucsd.edu

$$\tilde{T}_i(2\tau_T) = \tilde{T}_i(\tau_T) + \int_{\tau_T}^{2\tau_T} \dot{\tilde{T}}_i(t)dt \approx \left(1 + \zeta\overline{V_{i,2}^2} + \nabla^2\right)\tilde{T}_i(\tau_T) \equiv \hat{L}_1\hat{L}_0 T_i(0)\,. \tag{A6}$$

Above, we've defined $\overline{V_{i,l}^2}$ to be the average of V_i^2 over a time interval $[(l-1)\tau_T, l\tau_T)$ (since $\tau_V << \tau_T$, V_i^2 is not approximately constant over intervals of length τ_T, and there is no need to distinguish between V_i and $\tilde{V}_i$). We introduce the operator $\hat{L}_p$, which time-evolves $\tilde{T}_i(p\tau_T)$ to $\tilde{T}_i((p+1)\tau_T)$, for compactness. This generalizes to

$$\tilde{T}_i(l\tau_T) \approx \left(\prod_{p=0}^{l-1} \hat{L}_p\right) T_i(0)\,. \tag{A7}$$

For $l \geq 2$, notice that $\tilde{T}(l\tau_T)$ will have terms $\sim \nabla^2\overline{V_{i,l-1}^2}$ to $\sim \nabla^2 \ldots \nabla^2\overline{V_{i,1}^2}$, the latter of which has $l-1$ ∇^2's. These terms implicitly depend on V_j through the diffusive coupling, where j can range from the 1st to $(l-1)^{\text{th}}$ nearest-neighbor of the ith site. Applying a similar technique for $\tilde{V}_i$,

$$\tilde{V}_i(\tau_T) = V_i(0) + \int_0^{\tau_T} \dot{\tilde{V}}_i(t)dt \approx V_i(0) - \frac{\tau_T}{\tau_V}\frac{1}{T_c}\left(T_i(0)\overline{V_{i,1}^2}\right)\,, \tag{A8}$$

$$\tilde{V}_i(2\tau_T) \approx \tilde{V}_i(\tau_T) - \frac{\tau_T}{\tau_V}\frac{1}{T_c}\left(\tilde{T}_i(\tau_T)\overline{V_{i,2}^2}\right) \approx V_i(0) - \frac{\tau_T}{\tau_V}\frac{1}{T_c}\left(T_i(0)\overline{V_{i,1}^2} + \tilde{T}_i(\tau_T)\overline{V_{i,2}^2}\right)\,. \tag{A9}$$

Again, this generalizes for $\tilde{V}_i(N\tau_T)$:

$$\tilde{V}_i(N\tau_T) \approx V_i(0) - \frac{\tau_T}{\tau_V}\frac{1}{T_c}\sum_{l=0}^{N-1} \tilde{T}_i(l\tau_T)\overline{V_{i,l+1}^2}\,. \tag{A10}$$

As is suggested by Eq. (A7), $\tilde{T}_i(l\tau_T)$ will implicitly depend on voltages $l-1$ sites away from the ith lattice site. Since there is feedback between V_i and T_i, this means that $\tilde{V}_i(N\tau_T)$ will also depend on other V_j in a highly non-local manner. Additionally, after a sufficiently large time $N\tau_T$, these non-local couplings will have arbitrarily large order (the number of V_j which multiply one another). This spatial non-locality, induced by the interplay between V_i-T_i couplings and thermal site diffusion, is indicative of the LRO that we observe numerically.

Appendix B: Experimentally measured thermal interactions

Here, we provide some additional experimentally measured interactions between two neighboring thermal neuristors. Despite only having two neuristors, the interactions observed here offer valuable insights into the various phases of the thermal neuristor array.

In Fig. 5, we show the current-time curves of two adjacent neuristors under varying input voltages. Note that these devices are different from those discussed in the main text, possessing distinct threshold voltages, though their qualitative behaviors are consistent.

In panels (a) to (c) of Fig. 5, neuristor B is set with a 2.6 V input voltage, slightly below the threshold for independent spiking. However, the influence of neuristor A initiates spiking activities. Panel (a) shows that a 2.6 V input to neuristor A, also below its spiking threshold, results in mutual heating and a single synchronized spike in both neuristors. Increasing A's driving voltage leads to a stable spike train in A, as seen in panel (b), which in turn induces stable oscillations in B, forming a 2:1 spiking pattern. Further increasing A's voltage disrupts this synchronization, resulting in only a single spike in B, as depicted in panel (c).

Conversely, in panels (d) to (f), neuristor B operates under a 4.1 V input, capable of sustaining stable oscillations alone but sensitive to additional stimuli. Panel (d) shows that an optimal input to A yields a 1:1 synchronized pattern. However, a slight increase in A's voltage, as shown in panel (e), introduces phase lags between A and B. Eventually, as illustrated in panel (f), the significant phase lag disrupts B's stable spiking pattern, causing it to cease spiking.

In this example, both boundaries of the spiking thresholds exhibit first-order phase transitions, where minor changes in external stimuli lead to markedly different behaviors. This underpins the phase boundaries identified in Fig. 3 in the main text. Slight perturbations are amplified and propagated through the lattice, resulting in the diverse phase structures and LRO we observed.

Appendix C: Additional numerical results

1. Spiking patterns and avalanche size distributions under different conditions

In this section, we provide an intuitive understanding and visualization of spiking patterns and their corresponding avalanche size distributions under various conditions. The settings used here are consistent with those in Fig. 2 and Fig. 3 from the main text, and the figures presented retain the same meanings as those referenced.

First, to elucidate the phase structures depicted in Fig. 3 from the main text, Fig. 6 illustrates the avalanche size distributions and corresponding spiking patterns at a point within the rigid phase ($V^{\text{in}} = 12.5$ V, $C_{\text{th}} = 1$) and at a point near the no activity phase ($V^{\text{in}} = 13$ V, $C_{\text{th}} = 1.25$).

Furthermore, to highlight the significance of thermal coupling, Fig. 7 compares spiking patterns with and without thermal coupling at $V^{\text{in}} = 12$ V and $C_{\text{th}} = 1$. Given

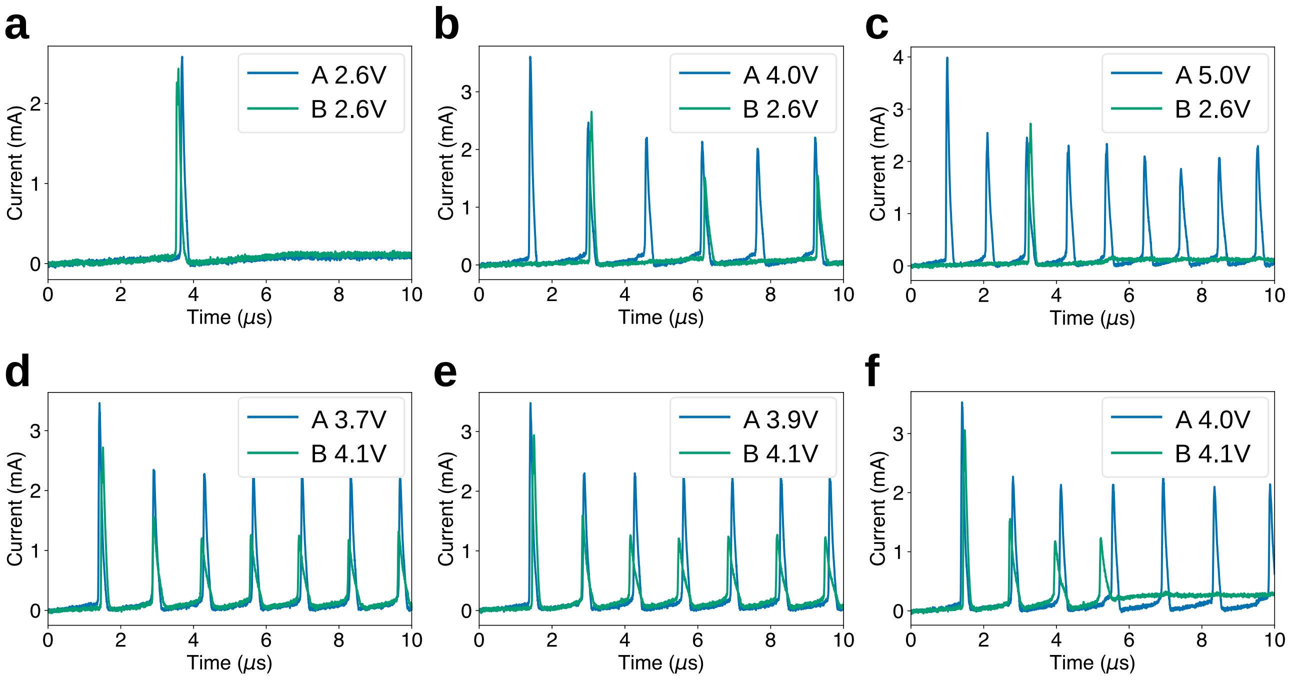

FIG. 5. Experimental demonstration of thermal interactions between two adjacent thermal neuristors. Panels (a)-(c) feature neuristor B under a 2.6 V input, just below its lower spiking threshold. Panels (d)-(f) depict neuristor B with a 4.1 V input, marginally below the upper spiking threshold. (a) A 2.6 V input to neuristor A triggers a single synchronized spike in both neuristors. (b) Increasing A's input to 4 V establishes a stable 2:1 spiking pattern. (c) Further increasing A's input to 5 V disrupts the synchronization, resulting in only one spike in B. (d) A 3.7 V input to A leads to 1:1 stable spiking oscillations. (e) A slight increase in A's input voltage introduces phase lags in the synchronization. (f) A further increase in A's voltage breaks the synchronization, causing B to stop spiking.

that thermal coupling is the only means through which neuristors exchange information, the absence of thermal coupling naturally results in random, uncorrelated spikes.

Additionally, in Fig. 8, we compare the spiking patterns and avalanche size distributions under periodic and open boundary conditions at $V^{\text{in}} = 9.96$ V and $C_{\text{th}} = 1$. This comparison shows that the type of boundary condition has minimal impact on the spiking behaviors, demonstrating the robustness of the spiking dynamics against boundary effects.

2. Finite-size scaling

In the main text (Fig. 3), we observed power-law distributions of avalanches across various system sizes, with closely matched power-law exponents at specific parameter points.

According to finite-size scaling theory [4], at criticality, avalanche size distributions for different system sizes should conform to a common scaling rule:

$$P(s, N) \sim s^{\alpha} \exp\left(-s/N^{\beta}\right), \tag{C1}$$

where s is the avalanche size, N is the system size, α is the critical exponent of the avalanche size distribution, and β is the cutoff exponent. As emphasized in several studies, such as [5, 6], the rescaling of avalanche size distributions according to Eq. (C1) should result in all distributions collapsing onto a single curve at criticality, characterized by a near-perfect overlap of all rescaled curves. Indeed, this phenomenon is typical in scale-free systems, with examples of near-perfect rescaling plots documented in references like [5–7].

Fig. 9 attempts this rescaling for the distributions presented in Fig. 3 from the main text. Here, we optimized the exponent β to align the four curves corresponding to different system sizes. However, our findings show a clear departure from Eq. (C1), suggesting the absence of scale-invariance and criticality.

3. Effect of noise strength

In the main text, we set the noise strength σ in Eq. (2) to 1 μJ$\cdot$s$^{-1/2}$. This section examines how varying the noise strength influences the phase structures of the system.

Fig. 10(a) presents the phase diagram for a 32×32 thermal neuristor array, exploring the interplay between thermal capacitance and noise strength. Here, the input

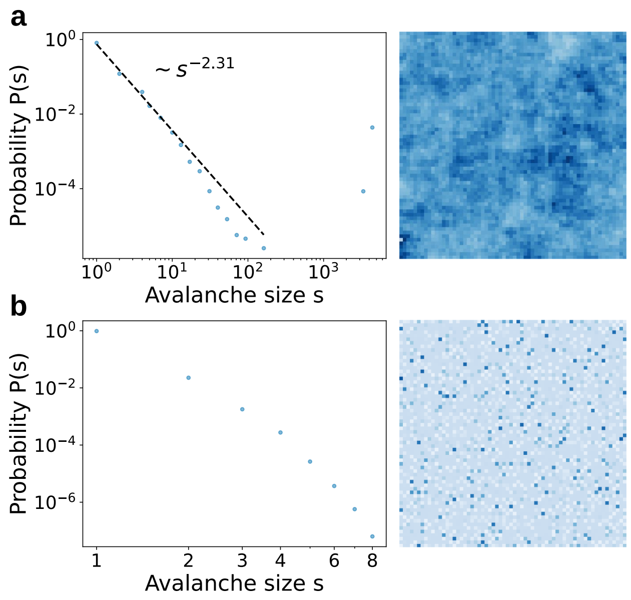

FIG. 6. Examples of avalanche size distributions and corresponding spiking patterns for settings consistent with Fig. 2 and Fig. 3 in the main text: (a) $V^{\rm in} = 12.5$ V, $C_{\rm th} = 1$. This setting corresponds to the rigid phase as depicted in Fig. 3(c). The right panel illustrates synchronized spiking behavior across nearly all neuristors, with the avalanche size distribution showing a power-law for smaller events. Notably, a prominent peak at the right end of the distribution indicates the prevalence of system-wide avalanches. (b) $V^{\rm in} = 13$ V, $C_{\rm th} = 1.25$. This setting is at the boundary of the "no activity" phase. The snapshot reveals random, uncorrelated spikes, and the avalanche size distribution is predominantly characterized by single or very small events.

voltage is consistently held at 12 V, while all other parameters remain as described in the main text. Fig. 10(b) plots the slopes of the avalanche size distributions for each set of parameters, paralleling the approach of Fig. 3 from the main text.

It is evident that noise strength significantly impacts the phase structures in this model. Near zero noise strength, when all neuristors are identical and begin with the same initial conditions, a rigid phase emerges across most parameters. This phase is characterized by synchronized spiking in all neuristors. Increasing the noise strength reveals a phase of LRO, situated between the rigid phase and the inactive phase. As the noise strength further escalates, the rigid phase vanishes, giving way to a disordered phase. In this phase, correlations between neuristors fade exponentially, resulting in isolated, uncorrelated spikes. In our main text simulations, we chose a noise strength of 1 μJ$\cdot$s$^{-1/2}$, leading to a wide variety of oscillation patterns.

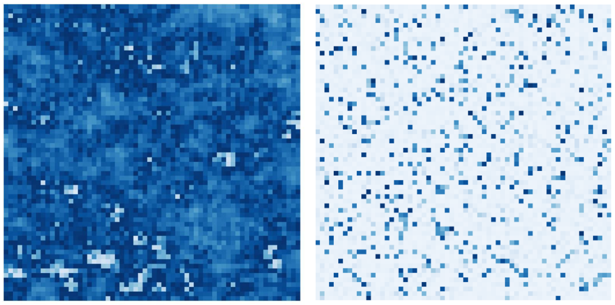

FIG. 7. Comparison of spiking patterns with (left) and without (right) thermal coupling at $V^{\rm in} = 12$ V, $C_{\rm th} = 1$. With thermal coupling, the setting induces a rigid state where almost all neuristors spike simultaneously, demonstrating highly correlated behavior. Conversely, without thermal coupling, the neuristors spike independently, resulting in uncorrelated, isolated spiking patterns.

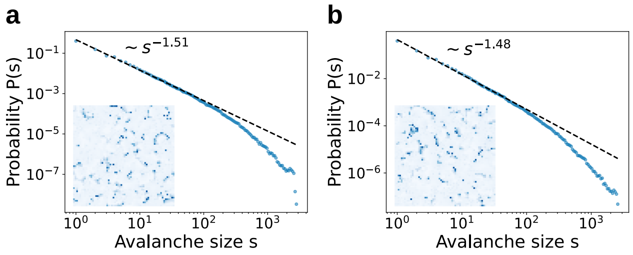

FIG. 8. Comparison of spiking patterns and avalanche size distributions under (a) periodic and (b) open boundary conditions at $V^{\rm in} = 9.96$ V and $C_{\rm th} = 1$, both settings within the LRO phase. The figure demonstrates that the type of boundary condition has minimal impact on the spiking behaviors and avalanche size distributions, indicating robustness of the spiking dynamics against boundary effects.

4. Hyperparameter selection in reservoir computing

Hyperparameter tuning is a critical aspect of machine learning algorithms. In our study, the parameters of the thermal neuristor array serve as hyperparameters for the reservoir computing algorithm.

As detailed in the main text, we use a 28×28 array of thermal neuristors for handwritten digit recognition on the MNIST dataset. While Bayesian optimization algorithms can efficiently select hyperparameters [8], we opt for a grid search to gain deeper insight into the reservoir's physics. Fig. 11 illustrates the accuracy of predictions after training for one epoch. In panel (a), we adjusted the thermal capacitance $C_{\rm th}$ and the input voltage for black pixels ($V_{\max}$), while keeping the input voltage for white background pixels ($V_{\min}$) at 10.5 V, and the noise strength σ at 0.2 μJ$\cdot$s$^{-1/2}$. Similarly, in panel (b), we fixed $V_{\min} = 10.5$ V and $V_{\max} = 12$ V, and varied σ and $C_{\rm th}$.

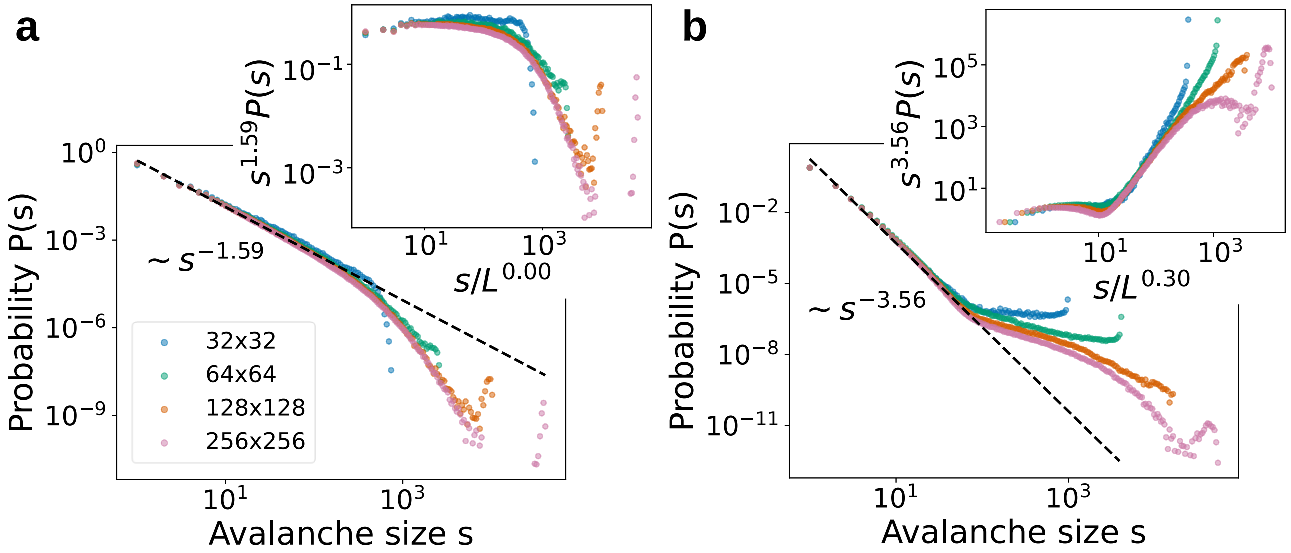

FIG. 9. Rescaling of the avalanche size distributions from Fig. 3 in the main text, based on the finite-size scaling ansatz, Eq. (C1). In the inset, $L = \sqrt{N}$ is the length of the lattice. Criticality should result in an overlap of curves from different system sizes, demonstrating scale-invariance. However, this scale-invariance is notably absent in our system, suggesting a lack of criticality.

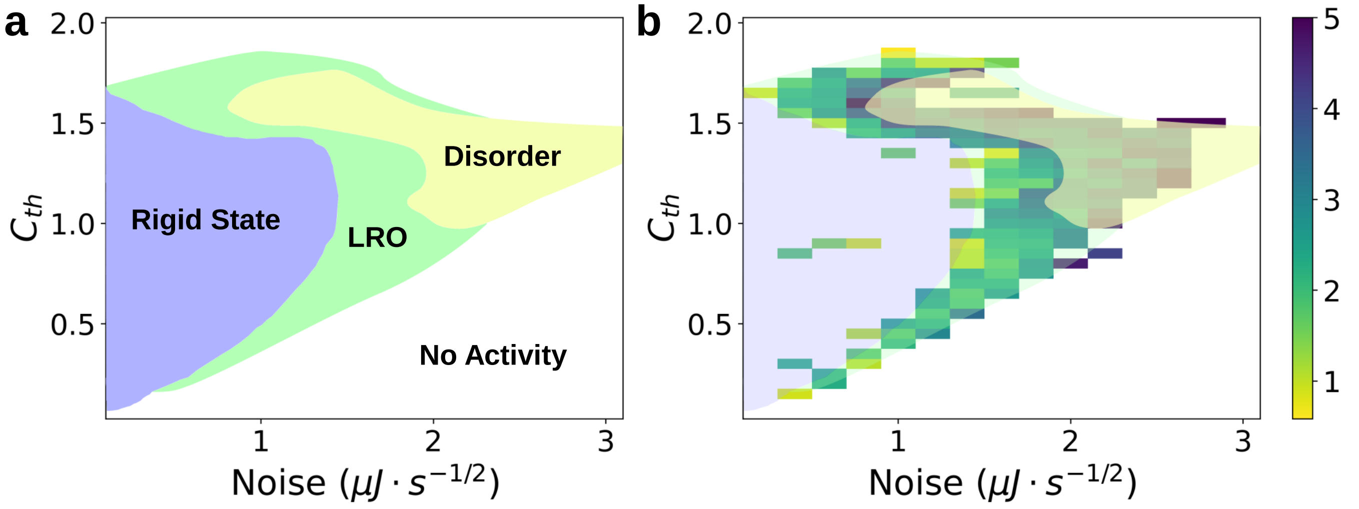

FIG. 10. Phase diagram of a 32 × 32 thermal neuristor array under varying noise strength and thermal capacitance, with a fixed input voltage of 12 V. As noise strength approaches zero, a rigid state prevails, characterized by synchronized firing of all neuristors. With increasing noise, a phase of LRO emerges, eventually giving way to a disordered phase at higher noise levels, characterized by uncorrelated spiking. (b) The slope of the avalanche size distributions at each parameter point, capped at 5 and excluding the negative sign for clarity. A lack of color in a box signals an unsuccessful power-law fit. The phase diagram from panel (a) is overlaid for context.

In both panels, most parameter combinations yield reasonable performance, provided some level of spiking dynamics is present in the system. The best performance is achieved near the phase transition boundary between no activity and the rigid state, with a small thermal capacitance ($C_{\rm th} \sim 0.15$), moderate input voltage ($V_{\rm max} \sim 12$ V), and small noise strength ($\sigma \sim 0.2$ μJ$\cdot$s$^{-1/2}$). A lower thermal capacitance facilitates a quicker response in the thermal neuristor array, while a smaller noise makes the system more predictable, enhancing the classification task.

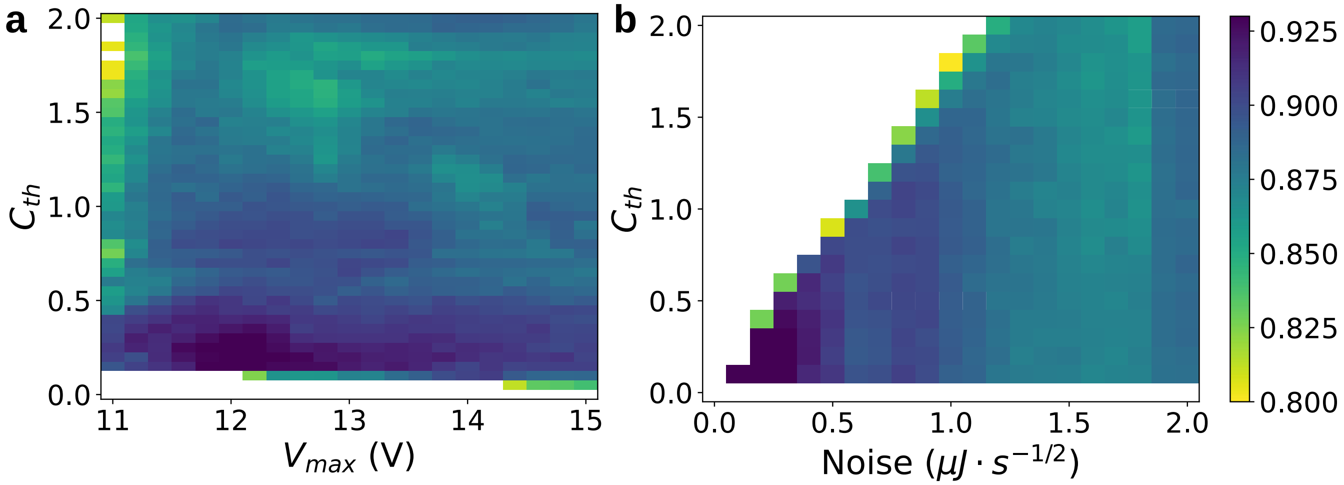

FIG. 11. Classification accuracy on the MNIST dataset after one epoch of training with various parameters. Areas in white indicate regions lacking spiking dynamics. (a) Varying the maximum input voltage V_{max} and thermal capacitance C_{th}, while fixing the minimum input voltage V_{min} to be 10.5 V and the noise strength σ to be 0.2 μJ$\cdot$s$^{-1/2}$. Optimal performance is observed at the point $C_{\text{th}} = 0.15$, $V_{\text{max}} = 12.2$ V. (b) Fixing $V_{\text{min}} = 10.5$ V and $V_{\text{max}} = 12$ V. Optimal performance is observed at the point $\sigma = 0.2$ μJ$\cdot$s$^{-1/2}$ and $C_{\text{th}} = 0.1$.

5. Further analysis of LRO in reservoir computing

In the preceding section, we determined that the optimal parameters place us within the synchronized rigid phase, as depicted in Fig. 10. However, this configuration does not necessarily imply that the reservoir operates in a rigid state, given that variations in the input data may introduce additional structures and correlations within the reservoir.

To rigorously test this hypothesis and explicitly quantify LRO within the reservoir, we computed the avalanche size distribution when the MNIST dataset served as the input. The experimental setup adheres to the methodology outlined in the main text, employing identical parameters. We recorded the current spikes to calculate the avalanche size distribution using the approach detailed in the Methods section from the main text.

The avalanche size distribution, encompassing all 60,000 training images from the MNIST dataset, is depicted in Fig. 12(c). Although the system is in a rigid state, the distribution reveals long-range structures: a power-law distribution is evident for smaller clusters up to $s \sim 10^2$, followed by two prominent bumps, which correspond to avalanches that span across the entire system and those limited to the black pixels comprising the digits.

Superficially, the distinct structure observed within the MNIST dataset's avalanche size distribution may seem to suggest that LRO plays a crucial role in the classification of MNIST images. Instead, we now demonstrate that the emergence of these long-range structures originates from the dataset itself, rather than the reservoir, and that long-range correlations are not essential for effective performance in this task.

First, we eliminated all interactions within the reservoir by disconnecting the thermal coupling between adjacent neuristors. Consequently, each thermal neuristor responds solely to its designated input pixel, devoid of any contextual awareness. After fine-tuning the hyperparameters via Bayesian optimization [8] to maximize classification accuracy, we observed the resultant reservoir dynamics, as depicted in Fig. 12(a). The corresponding avalanche size distribution is illustrated in Fig.12(c), and an animation of these dynamics is available in Supplementary Movie 3. In this configuration, the distribution reveals more defined structures: system-wide avalanches represent the white background, while smaller avalanches delineate the fractures within the digits. Given the absence of inter-neuristor interactions, it is evident that these structures are derived *solely* from the dataset. When compared to the original distribution involving interactions, it becomes apparent that internal interactions within the reservoir tend to smooth out smaller avalanches while retaining the principal structures inherent to the dataset.

Upon training the output layer, the classification accuracy achieved on the MNIST dataset's test set is presented in Fig. 12(d). Remarkably, even in the absence of interactions within the reservoir, we attained an accuracy of 95.8%, which is nearly equivalent to the performance under the optimized setup that included interactions. This outcome substantiates the assertion that LRO within the reservoir is not essential for achieving high computational effectiveness.

In another experiment, we aimed to minimize LRO in the neuristor array as much as possible. Previous findings, as discussed in the main text, suggest that LRO is influenced by the separation of time scales. To address

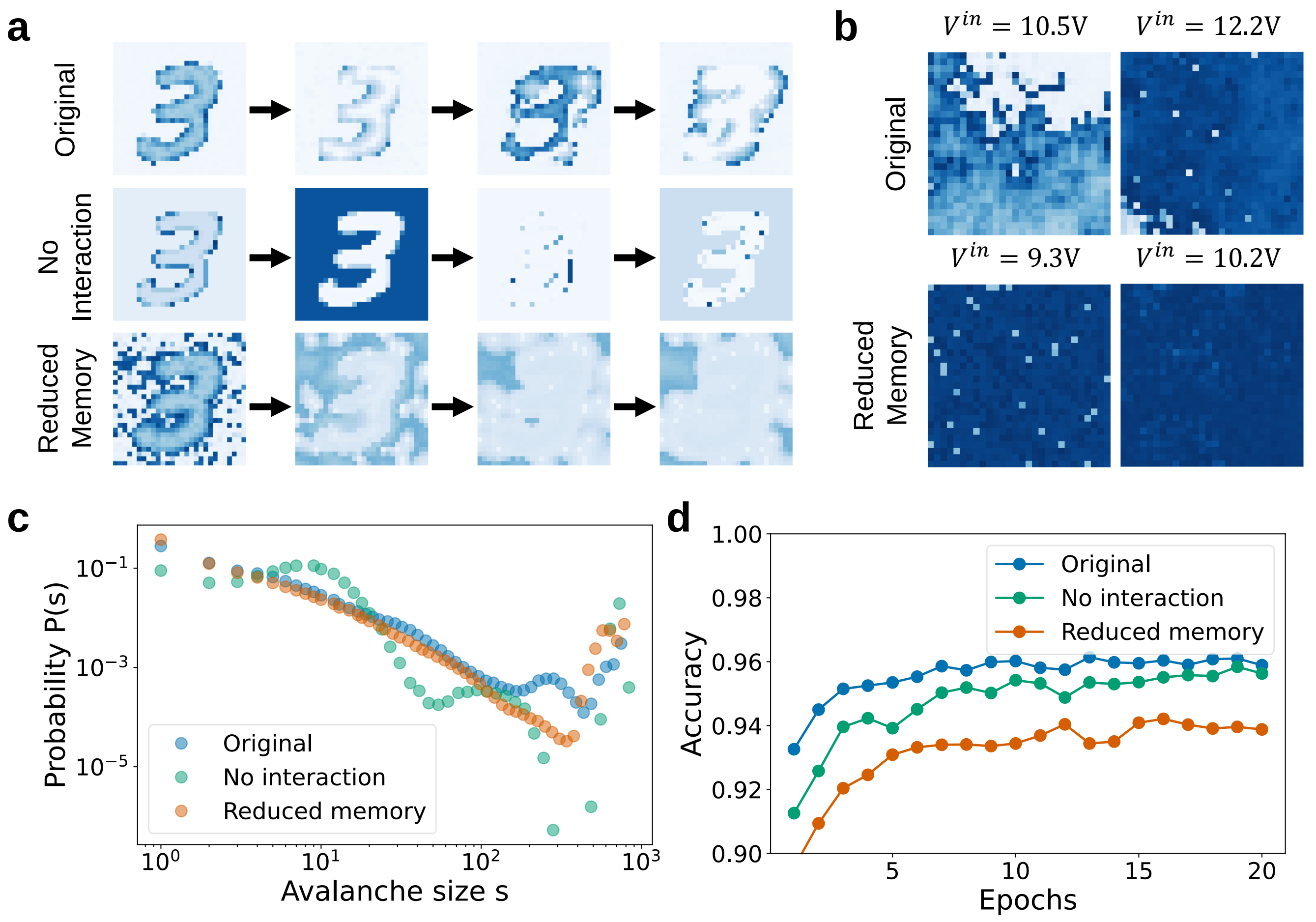

FIG. 12. Comparison of MNIST handwritten digit classification using reservoir computing under three different settings. **Original:** Described in the main text with parameters set to $V_{\min} = 10.50$V, $V_{\max} = 12.20$V, $C_{\rm th} = 0.15$, $\sigma = 0.2\mu{\rm J}\cdot{\rm s}^{-1/2}$. **No interaction:** Thermal interactions between neighboring neuristors are removed. Parameters are adjusted to $V_{\min} = 11.99$V, $V_{\max} = 13.37$V, $C_{\rm th} = 0.18$, $\sigma = 0$, and $S_c = 0$, replacing the original 4.11μW/K. **Reduced memory:** The slower time scale is minimized by raising the ambient temperature. The parameters are modified to $V_{\min} = 9.30$V, $V_{\max} = 10.16$V, $C_{\rm th} = 0.15$, $\sigma = 0.2\mu{\rm J}\cdot{\rm s}^{-1/2}$, and $T_0 = 330$K, up from 325K. Each setting is optimized to achieve the highest possible classification accuracy for the conditions specified. **(a) Snapshots of Reservoir Dynamics:** This panel showcases reservoir dynamics under three distinct settings, with each snapshot taken approximately 4μs apart. In the original setting, the parameters align the reservoir within the rigid phase, leading the digit pattern to evolve into propagating waves. In the no-interaction scenario, each neuristor's spiking is solely influenced by its individual input voltage, resulting in synchronized spiking across the white background, while pixels corresponding to the digits gradually desynchronize due to minor color variations. Under reduced memory, the reservoir quickly loses detailed information about the digit's shape, leading to a rapid degradation of recognizable patterns. **(b) Dynamics under Uniform Input:** This panel displays reservoir dynamics under uniform input voltages set to $V_{\min}$ and $V_{\max}$, confirming that the reservoir operates within a rigid state. The no interaction setting is omitted here, as all neuristors respond identically under uniform voltage conditions. **(c) Avalanche Size Distribution:** Presented here is the avalanche size distribution of current spikes using the MNIST dataset's 60,000 training images as input. In the no-interaction setting, the distribution mirrors the structural characteristics of the dataset, with system-wide avalanches representing the white background and smaller, digit-wide avalanches marking the black pixel areas. With interactions, the distribution for smaller avalanches follows a power-law, smoothing transitions seen in the non-interacting setup, while the configuration of larger avalanches remains intact. **(d) Classification Accuracy:** This panel reports classification accuracy on the MNIST test set after 20 epochs of training. The original configuration, as described in the main text, achieves the highest accuracy at 96.1%. Remarkably, the no-interaction scenario still performs well, achieving 95.8% accuracy without any internal information transfer within the reservoir. With reduced memory, where the slower time scale is lessened, long-range order within the system is substantially reduced, leading to a lower accuracy of 94.2%. These results further illustrate that there is no straightforward correlation between LRO and computational performance.

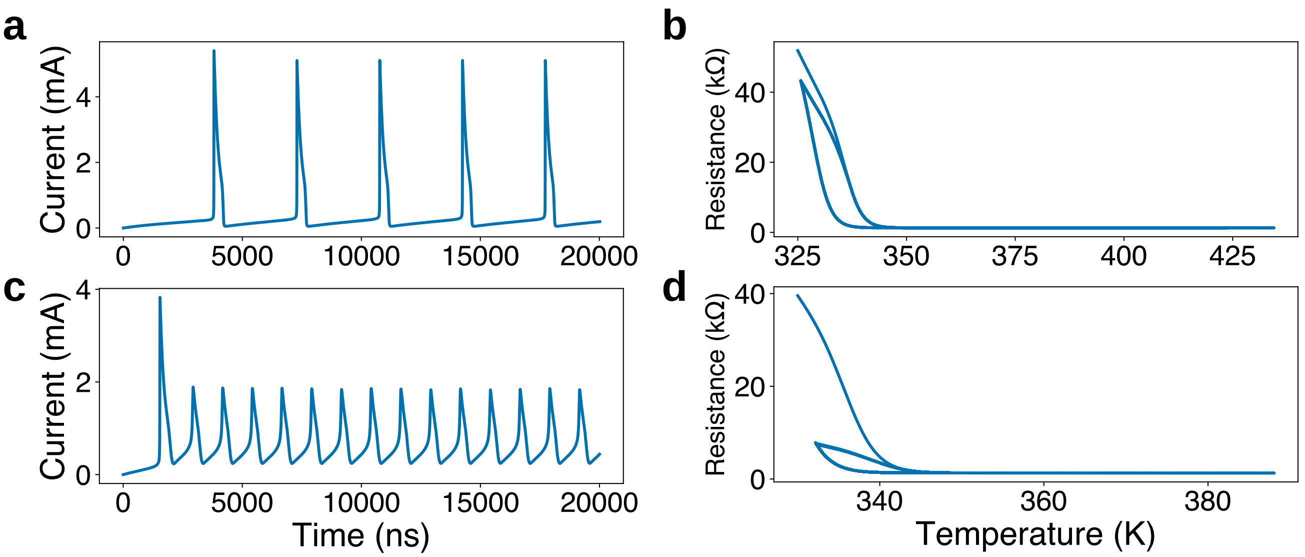

FIG. 13. Demonstration of how increasing ambient temperature, T_0, reduces the slower time-scale. (a) Spiking dynamics and (b) the resistance-temperature curve of a single neuristor at input voltage $V^{\text{in}} = 10.2\text{V}$, $C_{\text{th}} = 0.15$, and $T_0 = 325\text{K}$. The dynamics are stable, with the VO_2 device consistently reverting to its insulating state at $R_{\text{ins}} = 43\text{k}\Omega$ after each spike. (c) Spiking dynamics and (d) the resistance-temperature curve for the same neuristor under identical settings but with the ambient temperature increased to $T_0 = 330\text{K}$. The elevated temperature results in the VO_2 device reverting to a much lower resistance of $7\text{k}\Omega$ after each spike, significantly reducing the insulating RC time, τ_{ins}, to approximately $1\mu\text{s}$. This adjustment leads to smaller spiking amplitudes, a higher frequency, and more unstable spiking dynamics. Further increases in temperature or minor external perturbations can cause the VO_2 device to remain in its metallic state, ceasing to spike.

this, we attempted to reduce the memory within the system and eliminate the slower time scale by decreasing the resistance in the insulating state of the VO_2 device. This adjustment involved increasing the ambient temperature, T_0, from 325 K to 330 K. Fig. 13 illustrates the impact of this temperature increase on a single neuristor: the VO_2 device begins closer to the metallic state with reduced resistance, and after each spiking event, it resets to a lower resistance state. Further increase of T_0 ultimately leads the neuristors to remain in the metallic state, thereby ceasing to spike.

As illustrated in Fig.13(d), the maximum resistance in the insulating state during spiking, denoted as R_{ins}, is approximately $7\text{k}\Omega$. This value results in an insulating time constant, $\tau_{\text{ins}} = R_{\text{ins}}C \sim 1\mu\text{s}$, significantly reduced from $\tau_{\text{ins}} = 7.57\mu\text{s}$ mentioned in the main text. Consequently, both memory and LRO within the system are diminished. Furthermore, as shown in Fig.13(c), the spiking frequency increases, while the amplitudes of the spikes decrease. This alteration in dynamics leads to a spiking pattern that is more susceptible to disruption by external perturbations.

Once again, we optimized the hyperparameters using Bayesian optimization [8] to achieve the best classification accuracy on the MNIST dataset. Fig.12(a) captures snapshots of the reservoir dynamics, with an accompanying animation available in Supplemental Movie 4. These visuals demonstrate how the digit patterns rapidly blur and fade, indicating a rapid loss of memory within the system. Fig.12(b) shows that synchronized system-wide spikes from uniform input confirm the reservoir's operation in a rigid state under these conditions. However, when using the MNIST dataset as input, the avalanche size distribution, depicted in Fig.12(c), shows a power-law distribution for smaller avalanches and system-wide activities, mirroring the original setup. As noted previously, this pattern mostly originates from structural features inherent in the dataset, particularly since the modified reservoir struggles to maintain LRO. The classification accuracy recorded on the test set, as depicted in Fig.12(d), ultimately reaches 94.2%. Although the classification task is executed effectively, the neuristors' unstable spiking behavior remains less than ideal for achieving higher accuracy.

From the two experiments described above, it is clear that LRO may not necessarily emerge from the reservoir itself, rather it can be inherited from the dataset. Across all three experiments, there was no discernible correlation between LRO and computational performance. While the original setup, which included the possibility of LRO, performed the best, the non-interacting setup yielded nearly comparable results without any long-range interactions. Meanwhile, the experiment with reduced memory, although showing the lowest performance, still exhibited similar long-range structures as the original setup. Therefore, we conclude that LRO is not essential for ef-

fective computational performance in these scenarios.

6. Predicting chaotic dynamics with reservoir computing

In this section, we describe an experiment designed to predict chaotic dynamics governed by the 2D Kuramoto-Sivashinsky (KS) equations [9] using a reservoir computing framework implemented with a thermal neuristor array.

The 2D KS equation is expressed as:

$$\frac{\partial u}{\partial t} + \frac{1}{2}|\nabla u|^2 + \Delta u + \Delta^2 u = 0 \tag{C2}$$

where the boundary conditions are spatially periodic. This equation has been extensively studied [9] and is known for its chaotic behavior, which poses significant challenges in predicting long-term dynamics.

We discretized Eq. (C2) on a 16×16 square lattice with a unit bond length and simulated the dynamics numerically using a 4th order Runge-Kutta method with a time step of $\Delta t = 0.05$. The simulation began from random initial conditions and, after allowing for the decay of initial transients, the dynamics of the u field were recorded as training data.

To predict the chaotic dynamics described by the KS equation, we utilized reservoir computing with an array of thermal neuristors. This setup is similar to that used in the MNIST classification experiment detailed in the main text, allowing for a direct comparison of the techniques' effectiveness across different types of machine learning tasks.

In our approach, noting that the mean value of the u field is nonzero and decreases over time, yet the KS equation (C2) depends only on the gradient and not the magnitude of u [9], we performed a preprocessing step. This involved subtracting the mean from the u field and normalizing it between 0 and 1 to yield the transformed field, $\tilde{u}$. Snapshots of the $\tilde{u}$ field are shown in Fig. 14(a). Each $\tilde{u}(x, y)$ value is then linearly transformed into an input voltage for the corresponding thermal neuristor, with the spiking dynamics of the neuristor array serving as the output feature from the reservoir. An output layer is subsequently trained to predict the incremental change $u(x, y, t + \Delta t) - u(x, y, t)$.

Reservoir hyperparameters were optimized using the hyperopt library [8]. The optimized parameters included $C_{\text{th}} = 1.073$, noise strength $\sigma = 0$, and the transformation formula $V^{\text{in}} = (11.83 - 0.48\tilde{u})\text{V}$. Notably, the optimization yielded zero noise, underscoring that noise is detrimental in this reservoir computing task.

To enhance performance on this dataset, we implemented several modifications to the reservoir:

1. The output from the reservoir consists of the magnitudes of the current spikes, rather than merely indicating the presence of a spike.
2. Reflecting the local interaction nature of Eq. (C2), we replaced a fully-connected output layer with a convolution-like layer, where each output neuron is connected to the 5×5 nearest neighbors in the reservoir.
3. We eliminated the softmax nonlinearity in the output layer and employed linear regression for training.

This modified procedure predicts $u(x, y, t + \Delta t)$ from $u(x, y, t)$ at time t. Long-time predictions are iteratively performed by using the predicted u field as the input for the subsequent prediction step. The prediction results after 20 and 100 steps with $\Delta t = 0.05$ are illustrated in Fig. 14(a), and the mean-square-error of the prediction is plotted in Fig. 14(b), demonstrating good agreement with the ground truth. An example of the dynamics within the reservoir is depicted in Fig. 14(c), which loosely resembles the patterns in the $\tilde{u}$ field.

To verify the presence of LRO, we calculated the avalanche size distribution within the reservoir using the transformed $\tilde{u}$ field from the KS dynamics as input. The results, depicted in Fig. 14(d), are characterized predominantly by system-wide avalanches, indicating that the system maintains a rigid state even with non-uniform inputs. Moreover, the implementation of a locally connected output layer demonstrates that local information alone is sufficient for predicting dynamics, further reinforcing the notion that LRO is not necessary for achieving optimal computational performance.

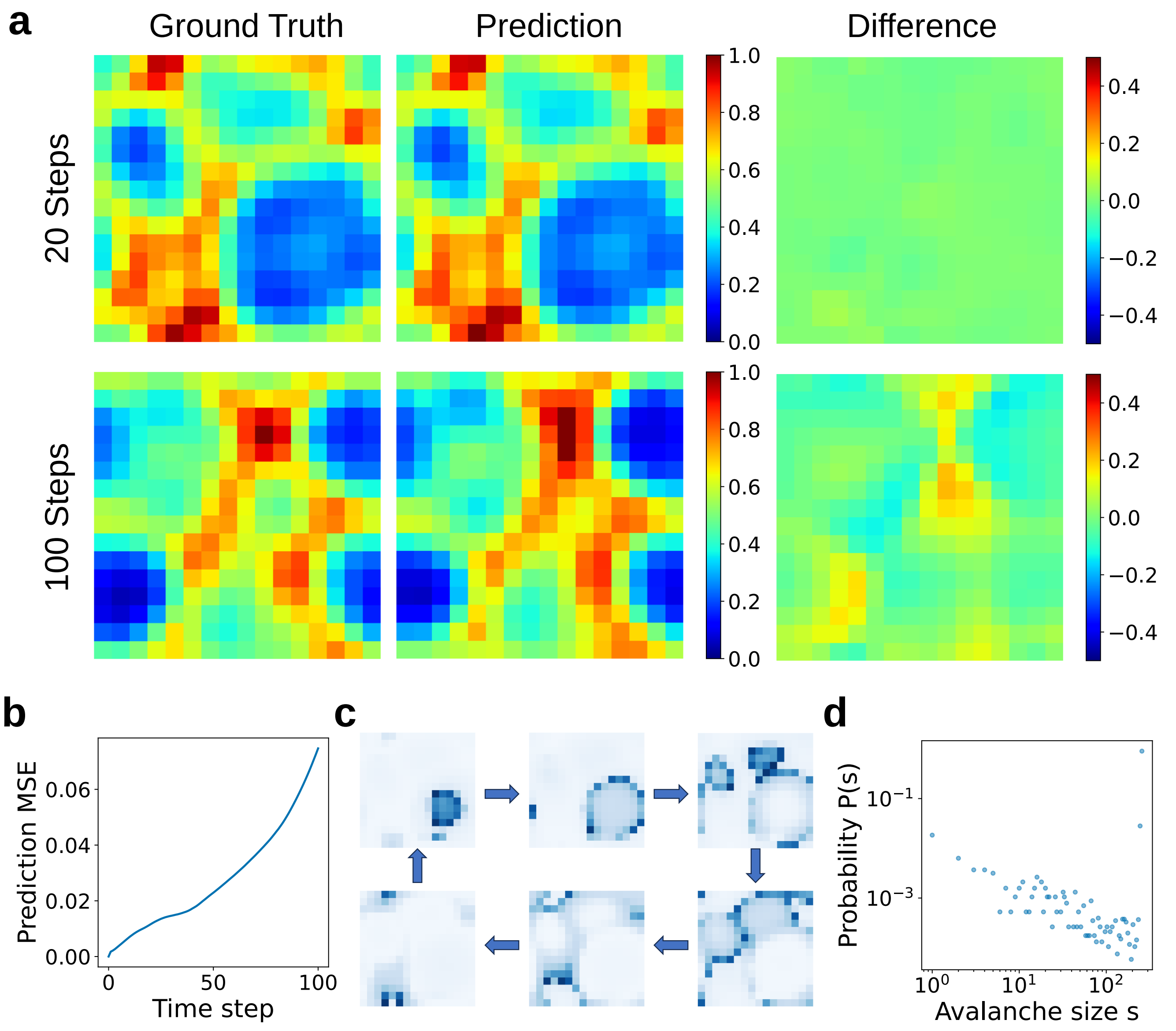

FIG. 14. Predicting the chaotic dynamics governed by the 2D Kuramoto-Sivashinsky (KS) equations using reservoir computing. (a) Comparison of the ground truths and predictions, after 20 and 100 time steps, with each time step corresponding to $\Delta t = 0.05$ (arbitrary unit). The two rightmost panels illustrate the differences between the predictions and the ground truths, highlighting the model's accuracy over time. (b) The mean-square-error (MSE) of the prediction as a function of the number of time steps predicted. Although the prediction MSE gradually increases with more extended simulation of the dynamics, the predictions remain reasonably accurate for up to 100 time steps, equivalent to $\Delta t = 5$ (arbitrary unit). (c) An example of reservoir dynamics is presented, showing patterns that loosely resemble the evolution observed in the KS equation dynamics. (d) Avalanche size distribution with KS dynamics as input to the reservoir. A prominent peak at the right end of the distribution highlights the predominance of system-wide avalanches, confirming that the reservoir operates in a rigid state.

[1] N. W. Ashcroft and N. D. Mermin, *Solid State Physics* (Saunders College Publishing, 1976).
[2] H. R. Wilson, Excitatory and inhibitory interactions in localized populations of model neurons, Biophysics Journal **12**, 1 (1972).
[3] J. G. Kirkwood, The Statistical Mechanical Theory of Transport Processes I. General Theory, The Journal of Chemical Physics **14**, 180 (1946).
[4] M. E. Fisher and M. N. Barber, Scaling theory for finite-size effects in the critical region, Physical Review Letters **28**, 1516 (1972).
[5] L. Brochini, A. de Andrade Costa, M. Abadi, A. C. Roque,

J. Stolfi, and O. Kinouchi, Phase transitions and self-organized criticality in networks of stochastic spiking neurons, Scientific reports **6**, 35831 (2016).

[6] J. A. Bonachela and M. A. Munoz, Self-organization without conservation: true or just apparent scale-invariance?, Journal of Statistical Mechanics: Theory and Experiment **2009**, P09009 (2009).

[7] C. Sipling and M. Di Ventra, Memory-induced long-range order in dynamical systems, arXiv preprint arXiv:2405.06834 (2024).

[8] J. Bergstra, D. Yamins, and D. Cox, Making a science of model search: Hyperparameter optimization in hundreds of dimensions for vision architectures, in *International conference on machine learning* (PMLR, 2013) pp. 115–123.

[9] A. Kalogirou, E. E. Keaveny, and D. T. Papageorgiou, An in-depth numerical study of the two-dimensional kuramoto–sivashinsky equation, Proceedings of the Royal Society A: Mathematical, Physical and Engineering Sciences **471**, 20140932 (2015).
