# Supplementary material for: Collective dynamics and long-range order in thermal neuristor networks
Source: Nat Commun. 2024 Aug 14;15:6986. doi: 10.1038/s41467-024-51254-4 (PMC11324871; doi:10.1038/s41467-024-51254-4)
Supplement: Supplementary file 3 — Description of Additional Supplementary Files [file 41467_2024_51254_MOESM3_ESM.pdf]

Description of Additional Supplementary Files

Supplementary Movie 1. Different oscillation patterns in a 64×64 array of thermal neuristors. This is a dynamic visualization of Fig. 2 in the main text.

Supplementary Movie 2. Collective oscillations when using images of handwritten digits as inputs to the thermal neuristor network. This is a dynamic visualization of Fig. 4 in the main text.

Supplementary Movie 3. Collective oscillations when using images of handwritten digits as inputs to the thermal neuristor network, with thermal interactions between neighboring neuristors removed. This is a dynamic visualization of Fig. 12(a) in the Supplementary Information.

Supplementary Movie 4. Collective oscillations when using images of handwritten digits as inputs to the thermal neuristor network, with the slower time scale within the network significantly reduced. This is a dynamic visualization of Fig. 12(a) in the Supplementary Information.
