# Supplementary figures and images for: Collective dynamics and long-range order in thermal neuristor networks
Source: Nat Commun. 2024 Aug 14;15:6986. doi: 10.1038/s41467-024-51254-4 (PMC11324871; doi:10.1038/s41467-024-51254-4)

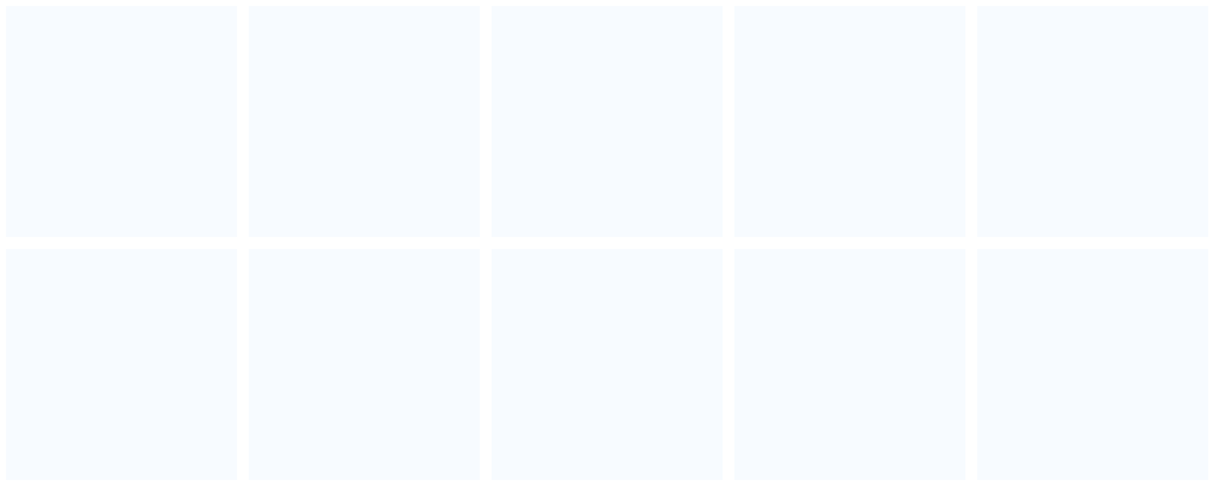

Supplement: Supplementary file 5 — Supplementary Movie 2 [file 41467_2024_51254_MOESM5_ESM.gif]

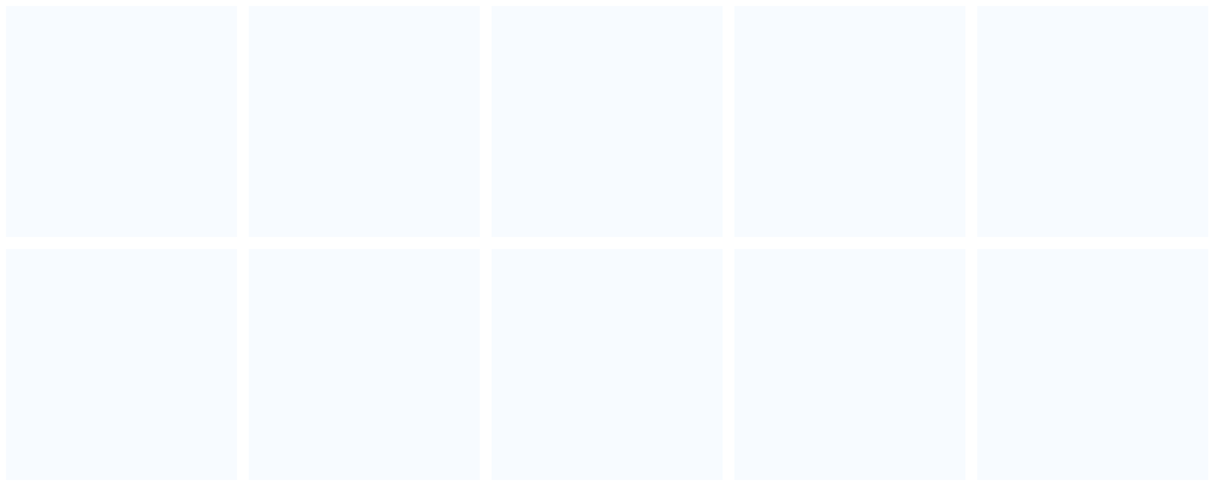

Supplement: Supplementary file 6 — Supplementary Movie 3 [file 41467_2024_51254_MOESM6_ESM.gif]

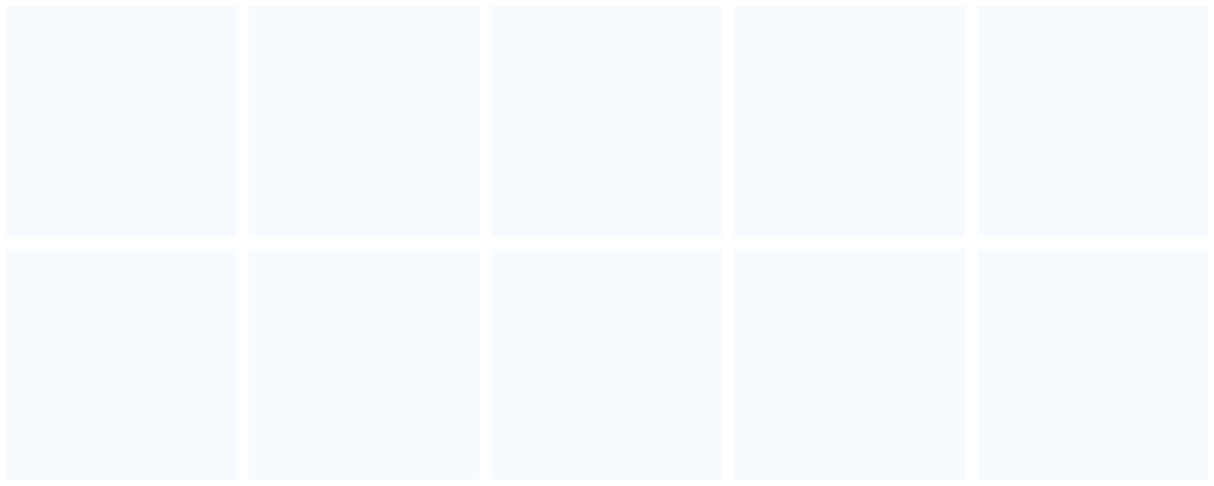

Supplement: Supplementary file 7 — Supplementary Movie 4 [file 41467_2024_51254_MOESM7_ESM.gif]
